# The Role of Gut Microbiota in Obesity and Type 2 and Type 1 Diabetes Mellitus: New Insights into “Old” Diseases

**DOI:** 10.3390/medsci6020032

**Published:** 2018-04-17

**Authors:** Igor Alexander Harsch, Peter Christopher Konturek

**Affiliations:** 1 Division of Endocrinology and Metabolism, Thuringia Clinic Saalfeld “Georgius Agricola”, Department of Internal Medicine II, Teaching Hospital of the University of Jena, Rainweg 68, D-07318 Saalfeld/Saale, Germany; 2 Division of Gastroenterology, Thuringia Clinic Saalfeld “Georgius Agricola”, Department of Internal Medicine II, Teaching Hospital of the University of Jena, Rainweg 68, D-07318 Saalfeld/Saale, Germany; pkonturek@thueringen-kliniken.de

**Keywords:** gut microbiome, obesity, metabolic syndrome, type 2 diabetes mellitus, type 1 diabetes mellitus, butyrate, probiotics, lipopolysaccharides, faecal microbiota transfer, metformin

## Abstract

The investigation of the human microbiome is the most rapidly expanding field in biomedicine. Early studies were undertaken to better understand the role of microbiota in carbohydrate digestion and utilization. These processes include polysaccharide degradation, glycan transport, glycolysis, and short-chain fatty acid production. Recent research has demonstrated that the intricate axis between gut microbiota and the host metabolism is much more complex. Gut microbiota—depending on their composition—have disease-promoting effects but can also possess protective properties. This review focuses on disorders of metabolic syndrome, with special regard to obesity as a prequel to type 2 diabetes, type 2 diabetes itself, and type 1 diabetes. In all these conditions, differences in the composition of the gut microbiota in comparison to healthy people have been reported. Mechanisms of the interaction between microbiota and host that have been characterized thus far include an increase in energy harvest, modulation of free fatty acids—especially butyrate—of bile acids, lipopolysaccharides, gamma-aminobutyric acid (GABA), an impact on toll-like receptors, the endocannabinoid system and “metabolic endotoxinemia” as well as “metabolic infection.” This review will also address the influence of already established therapies for metabolic syndrome and diabetes on the microbiota and the present state of attempts to alter the gut microbiota as a therapeutic strategy.

## 1. Introduction

Joshua Lederberg was a molecular biologist and geneticist who created the term “microbiome” in 2001 [1]. This term refers to the totality of microbes that colonize humans and their genes [2]. An adult human being is colonized by about 100 trillion microbes, predominantly in the gastrointestinal tract (GI). Harbouring these enormous numbers of microorganisms, the gut microbiota is sometimes referred to as a “hidden organ.” The human intestine hosts prokaryotes (bacteria, archaea), viruses and fungi. In the human GI tract, the largest number of microbiota reside in the colon.

*Firmicutes* such as the *Ruminococcus, Lactobacillus* and *Clostridium* species, as well as *Bacteroidetes (Bacteroidaceae, Prevotellaceae)* account for the largest proportion of intestinal microbiota. The term “*Firmicutes*” stems from the Latin words “firmis” meaning strong and “cutis” meaning skin. This classification is derived from the attempt to include all bacteria with a Gram-positive cell wall structure. However, newer insights have shown that a few of these bacteria do stain Gram-negative.

“*Bacteroidetes*” is the phylum that is composed of three classes of Gram-negative, non-spore-forming, anaerobic or aerobic, bacteria. They are widely distributed in the environment, including the soil, in sediments and in sea water, as well as in the gut and on the skin.

A problem with our understanding of the composition and function of the gut microbiota is that many of the microbial cells cannot be successfully cultured yet. Interesting new insights for the study of the microbiome, its composition, its influence on immunoregulation and other factors are revealed by methods of high-throughput sequencing of genomes in combination with gene expression analyses and mass spectrometric methods [3,4,5]. Our understanding of the composition and function of gut microbiota is rapidly evolving—a MEDLINE search for the terms “gut microbiota obesity” generates 2158 hits and “gut microbiota diabetes” 1419 hits (last accessed 5 April 2018).

In recent years, the growing insights into this complex ecosystem have often been generated from the study of germ-free (GF) animals, since compositional studies do not necessarily provide information on the functional potential of the different populations residing in the gut. These studies also enhanced our understanding concerning a possible role in the aetiology of several diseases. This especially accounts for possibly pathological alterations in the gut microbiota—termed dysbiosis.

## 2. Obesity and Type 2 Diabetes Mellitus

With this in mind, major public health issues such as obesity, metabolic syndrome and diabetes mellitus and their possible relation to the microbiome were amongst the focus of research. According to the National Expert Panel on Detection, Evaluation and Treatment of High Blood Cholesterol in Adults (NCEP-ATP-III) [6], the diagnosis of “metabolic syndrome” is made if at least three of the following five criteria are met:

- Abdominal fat distribution, determined by an abdominal circumference of over 102 cm in men or over 88 cm in women (Caucasian).
- Serum triglycerides greater than 150 mg/dL (>1.7 mmol/L), or therapy already initiated to reduce triglycerides.
- High density lipoprotein (HDL) cholesterol ≤ 40 mg/dL (<1.05 mmol/L) in men or <50 mg/dL (1.25 mmol/L) in women.
- Blood pressure of 130/85 mmHg or more, or already initiated therapy to reduce hypertension.
- Fasting blood sugar ≥ 110 mg/dL (5.6 mmol/L), or type 2 diabetes.

Obesity is a predisposing element of the metabolic syndrome in the development of type 2 diabetes mellitus (T2 DM). Obesity is a major risk factor for type 2 diabetes which accounts for 90–95% of all diabetes cases [7]. Numerous reviews on gut microbiota and metabolic disorders have been published so far. This review will focus on the following fields: Microbiota composition, pathophysiology and evolving therapeutic strategies in the 3 disorders; Obesity as a prequel to type 2 diabetes (sometimes referred to as “diabesity”); type 2 diabetes and type 1 diabetes (T1 DM).

The first part of this review focuses on the role of microbiota in obesity.

### 2.1. The Role and Composition of Gut Microbiota in Developing Elements of the Metabolic Syndrome with Special Regard to Obesity

Early studies aimed to clarify whether there are significant differences in the gut microbiome in lean and in obese subjects in animal models as well as in humans and investigated the possible relevance of these differences. The first studies were performed using laboratory mouse models, which have the advantage that many confounding factors such as the environment, diet and genotype are known or can be controlled.

Backhed et al. in 2004 [8] investigated adult GF C57BL/6 mice. These animals are protected against developing obesity caused by consuming a high-fat, high-sugar diet. It was demonstrated that colonizing the mice’s GI with normal microbiota harvested from the distal intestine (cecum) of conventionally raised obese animals produced a 60% increase in body fat content and insulin resistance within 14 days despite reduced food intake.

Furthermore, the authors could demonstrate that obesity affects the diversity of the gut microbiota [9,10]. They analysed 5088 bacterial 16S rRNA gene sequences from the cecal microbiota in genetically obese ob/ob mice, lean ob/+ and wild-type siblings, and their ob/+ mothers, all fed the same polysaccharide-rich diet. The authors could demonstrate that, compared with lean mice and regardless of kinship, ob/ob animals have a 50% reduction in the abundance of *Bacteroidetes* and a proportional increase in *Firmicutes*. These changes indicate that, in this model, obesity affects the diversity of gut microbiota in mice.

In an attempt to explain the impact of the different microbiota between lean and obese animals, the authors [10,11] speculated that the microbiota of the obese animals might be more efficient in extracting energy from their diet than the microbiota of the lean animals. By shotgun sequencing [3], the authors compared the microbiota of obese mice (ob/ob) with those of lean mice (ob/+). They could demonstrate that the ob/ob genome was enriched with environmental gene tags encoding enzymes involved in the initial steps in breaking down dietary polysaccharides that are otherwise indigestible. The enrichment concerns eight glycoside hydrolase families, which are capable of degrading dietary polysaccharides and starch. Furthermore, genes for encoding proteins importing products of these glycoside hydrolases, the so-called ATP-binding cassette (ABC) transporters, the metabolization (e.g., alpha and beta-galactosidases), and generating the end products of fermentation (butyrate and acetate) are significantly enriched in the microbiota of the ob/ob mice. That said, the authors concluded that these mice have a gut microbiome with an increased capacity for energy harvest.

In a landmark study, Ridaura et al. [12] were the first to transplant human faeces into GF mice. It was demonstrated that culture collections generated from human microbiota samples can transmit the donor phenotype of mice: they transplanted faecal microbiota from human adult female twin pairs discordant for obesity into germ-free mice fed low-fat mouse feed, as well as diets representing different levels of saturated fat and fruit and vegetable consumption. The “human obesity” could be transferred and the mice developed increased total body and fat mass, as well as obesity-associated metabolic phenotypes. In mice containing the lean co-twin’s microbiota, this prevented the development of increased body mass and obesity-associated metabolic phenotypes.

To define the effects of diet on lean (ln) and obese (ob) microbiota-mediated transmission of body composition and metabolic phenotypes, they constructed a diet made with foods that characterize diets representing the lower tertile of consumption of saturated fats and the upper tertile of consumption of fruits and vegetables. Significant differences in body composition were documented between ob/ob and ln/ln mice consuming this diet.

Additionally, the authors used co-housing to determine whether exposure of a mouse harbouring a culture collection from the lean twin could prevent development of the increased adiposity phenotype and microbiome-associated metabolic profile of a cagemate colonized with the culture collection from its obese co-twin, or vice versa (mice are coprophagic). Interestingly, ob mice exhibited a significantly lower increase in adiposity compared to control ob animals that had never been exposed to mice harbouring the lean co-twin’s culture collection.

These findings demonstrate that a donor phenotype can be transmitted into the phenotype of a recipient. However, diet still plays a central role in developing an obese or a lean phenotype [12].

The possible diversity of human gut microbiota and its impact on metabolic syndrome with special regard to lean and obese individuals had also been addressed by Le Chatelier et al. in 2013 [13]. The authors studied the human gut microbial composition in a population sample of 123 non-obese and 169 obese Danish individuals. Their approach was to compare the gene number across the total study sample of individuals, which showed a bimodal distribution of bacterial genes. They termed individuals with <480,000 genes ‘low gene count’ (LGC) and the others ‘high gene count’ (HGC). These had on average 380,000 and 640,000 genes, respectively, suggestive of a more “rich” microbiota and more diverse microbial communities. Forty-six genera differed significantly in abundance between the HGC and LGC individuals. At the phylum level, this shift resulted in a higher abundance of *Proteobacteria* and *Bacteroidetes* in LGC individuals versus increased populations of *Verrucomicrobia, Actinobacteria* and *Euryarchaeota* in HGC individuals. Interestingly, at the genera level, there is a contrast between the distribution of anti-inflammatory species, such as *Faecalibacterium prausnitzii*, which are more prevalent in HGC individuals. Potentially pro-inflammatory bacteria such as *Bacteroides* and *R. gnavus*—which are also associated with inflammatory bowel disease [14,15]—were found to be more frequent in LGC individuals. These findings are suggestive of the domination of potentially pro-inflammatory bacteria in obese subjects. The LGC subjects had more marked overall adiposity, insulin resistance and dyslipidaemia. The phenotype of the LGC subjects was also associated with increased serum leptin, decreased serum adiponectin, insulin resistance, increased levels of triglycerides, free fatty acids, decreased HDL-cholesterol and a more marked inflammatory phenotype (increased highly sensitive C-reactive protein). Obese individuals among the lower bacterial richness group also gained more weight over time. However, it needs to be pointed out that not all LGC subjects were obese and not all HGC subjects were lean. This reflects that there is still an impact of the “own” genome of the individual. The latter is the “human” part of the “metagenome”, a term used for the composite of the human genome and the genomes of the microbiota colonizing the human body.

In terms of the distribution of phylae associated with the obese phenotype, an increase in the phylum *Firmicutes* and a decrease in *Bacteroidetes* associated with obesity were also observed in some previous studies [16,17]. However, Schwiertz et al. [18] reported in a total of 98 subjects that the most abundant bacterial groups in the faeces of lean and obese subjects were the phyla *Firmicutes* and *Bacteroidetes*. The ratio of *Firmicutes* to *Bacteroidetes* changed in favour of the *Bacteroidetes* in overweight and obese subjects.

Interestingly, in a subgroup of subjects from the Le Chantelier study [19] (38 obese and 11 overweight individuals), dietary intervention (6-week energy restricted high-protein diet followed by a 6-week weight-maintenance diet) improved low gene richness and the clinical phenotypes, but seemed to be less efficient for inflammation parameters in individuals with lower gene richness.

### 2.2. The Role and Composition of Gut Microbiota in Developing Elements of the Metabolic Syndrome with Special Regard to Diabetes Mellitus Type 2

The above-mentioned studies demonstrated the possible significance of the composition of gut microbiota in obesity. As to be expected, the typically obesity-related T2 DM was soon another focus of research.

A first study by Larsen et al. [20] included 36 male adults. 18 subjects were diagnosed with diabetes type 2; they had a mean age of 56 years, and a mean body mass index (BMI) of 30. The other 18 controls had a mean age of 59 years, and a mean BMI of 28. The total bacterial counts were similar in the diabetic and the control group. By characterization of the intestinal microbiota by tag-encoded pyrosequencing, the authors demonstrated that the proportion of *Firmicutes* was significantly higher in the controls compared to the diabetic group. The phylum *Bacteroidetes* and *Proteobacteria* were somewhat but not significantly enriched in the diabetic group compared to controls.

The authors concluded that T2 DM is associated with compositional changes in the intestinal microbiota mostly apparent at the phylum and class levels. These results are in agreement with the recent evidence obtained for overweight persons by Schwiertz and colleagues [18] though they contradict other previously mentioned studies [13,16,17]. Given the a.m. findings, a positive correlation between ratios of *Bacteroidetes* to *Firmicutes* and BMI could be expected. The reverse tendency was observed, which was regarded as indicative that overweight and T2 DM are associated with different groups of the intestinal microbiota.

However, the study group was small and no information was given on the therapy of the patients with diabetes, a factor also likely to have an impact on the microbiota.

Qin et al. [21] performed a metagenome-wide association study in 345 Chinese T2 DM subjects. It was designed as a case-control study with non-diabetic controls. Using a shotgun-sequencing approach, the authors detected a “moderate” degree of dysbiosis in the diabetic subjects. This meant—in comparison to the controls—a decrease in the abundance of various butyrate producing bacteria, including *Clostridiales* sp. *SS3/4, Eubacterium rectale*, *Faecalibacterium prausnitzii*, *Roseburia intestinalis* and *Roseburia inulinivorans* (all these bacteria belong to the phyla *Firmicutes*). Furthermore, they identified more opportunistic pathogens such as *Bacteroides caccae*, *Clostridium hathewayi*, *Clostridium ramosum*, *Clostridium symbiosum*, *Eggerthella lenta* and *Escherichia coli*. At the pathway level, the gut microbiota of T2 DM patients was functionally characterized by an enrichment in the membrane transport of sugars, branched-chain amino acid (BCAA) transport, methane metabolism, xenobiotics degradation and metabolism, and sulphate reduction. This does to some degree support the concept of an increased capacity for energy harvest proposed by Turnbaugh [10]. By contrast, there was a decrease in the level of bacterial chemotaxis, flagellar assembly, butyrate biosynthesis and metabolism of cofactors and vitamins. Comparable changes in intestinal bacteria composition have recently been reported for ageing people [22]. The findings may also suggest a protective role for butyrate-producing bacteria against T2 DM [23].

In a comparable approach, Karlsson et al. [24] investigated the faecal microbiota in 145 70-year old women—53 women had T2 DM, impaired glucose tolerance was present in 49 women, and 43 had normal glucose tolerance (NGT). In contrast to previous reports on observations between lean and obese people, the faecal microbiota of non-diabetic, impaired glucose tolerance (IGT) and T2 DM women contained similar numbers of genes. In the diabetic group, the authors observed an increase in the abundance of four *Lactobacillus* species and decreases in the abundance of five *Clostridium* species. As for metabolic control, *Lactobacillus* species correlated positively with fasting glucose and glycated hemoglobin (HbA1c), whereas *Clostridium* species correlated negatively with fasting glucose, HbA1c and other metabolic markers. Both species were in no correlation with the BMI of the test subjects. In a metagenomic cluster model, they identified *Roseburia* and *Faecali bacterium prausnitzii* as highly discriminant for T2 DM. These bacteria are known as human gut colonizers and butyrate producers. Such bacteria have been reported to improve diabetic control and insulin sensitivity [23], aspects that will be discussed later in this review.

In accordance with the study of Qin [21] and Karlsson [24] that supports the concept of an “increased capacity for energy harvest”, the non-diabetic and the T2 DM bacterial communities had different functional compositions and several pathways were differentially abundant in T2 DM and NGT women. These pathways which showed the highest scores for enrichment in T2 DM metagenomes included starch and glucose metabolism, fructose and mannose metabolism and ABC transporters for amino acids, ions and simple sugars.

As to be expected in different ethnicities and in those with different nutritional habits, many metagenomic markers for T2 DM are different between the Chinese and the European cohort; however, in agreement with the previous study, they observed that *Clostridium clostridioforme* metagenomic clusters were increased whereas *Roseburia_272* was decreased in T2 DM metagenomes. As for medication possibly confounding the results, the authors report that only two of the species included in their statistical model were affected by the use of metformin (*Clostridium botulinum Bstr.Eklund 17B* and *Clostridium* sp. *7_2_43FAA*).

Shortcomings of the above-mentioned studies include the relatively low number of patients analysed, the lack of a gender balance and the matching of the cohorts (e.g., age, BMI). The use of proton pump inhibitors and previous antibiotic therapy was not reported. Possible effects of antidiabetic therapy are not addressed in the Qin study [21] and the “insignificant influence” of metformin in the Karlsson [24] study raises doubts, as several effects of metformin, as well as of acarbose, on gut microbiota are reported and will be discussed later [25,26,27,28,29]. It can be assumed that in the European cohort there was a widespread use of metformin. On the other hand, acarbose is extensively used with Chinese patients [25].

However, the observation that a certain composition of gut microbiota may have an important impact on the aetiology of T2 DM, as well as on its control, definitely represents a major upgrade in our understanding.

### 2.3. Possible Pathophysiologic Action of Gut Microbiota in the Development of Obesity and Diabetes Mellitus Type 2

These possible mechanisms and concepts overlap in the pathophysiology of obesity and T2 DM and are not always to be disentangled. For didactic reasons, they are introduced separately. Moreover, the arrangement of the following concepts and theories are not listed according to their importance. Most of them are derived from animal models and their significance in humans still remains to be clarified.

#### 2.3.1. Bacteria Producing Butyrate and Other Short-Chain Fatty Acids: Role in Energy Intake, Food Regulation and Insulin Sensitivity

Butyrate is a short-chain fatty acid. In the study by Qin et al. [21], a decrease of butyrate-producing bacteria such as *Roseburia intestinalis* and *F. prausnitzii* was observed in T2 DM patients compared to healthy controls. These findings were confirmed in the postmenopausal T2 DM women in the Karlsson study [24].

Dietary fibres containing complex plant polysaccharides, if soluble, can be digested by certain bacteria of the gut microbiota. The host’s capacities are too low for this digestion and the host is also not able to digest non-soluble polysaccharides such as cellulose [30]. Products of the digestion of soluble plant polysaccharides are short-chain fatty acids as butyrate, but also acetate and propionate. These short-chain fatty acids are not only an important energy source—they also exert important roles in the regulation of food and energy intake [23]. Already characterized mechanisms are the bindings on G protein-coupled receptors (GPR) GPR 41 and GPR 43 [31]. These are abundant in adipocytes, gut immune cells and gut epithelial cells [32,33], but to our present knowledge not in the liver and muscles. The activation of these receptors induces a secretion of Protein YY (PYY). PYY is a short peptide that is released in the ileum and colon that acts to reduce appetite with direct effects on the central nervous system. Furthermore, Glucagon-like Peptide 1 is another gut peptide released via GPR receptors and is acting in similar ways to PYY. In a study by Kimura et al. [31], these effects were brilliantly outlined in a murine model. In normal mice, GPR 43 *is* mainly expressed in the immune tissue and the white adipose tissue (WAT). The authors tested GPR 43-deficient mice. These mice are obese on a normal diet, whereas mice overexpressing GPR 43 specifically in adipose tissue remain lean even when fed a high-fat diet. The important effect of gut microbiota as deliverers of GPR activating short-chain fatty acids is proven by the finding that, raised under germ-free conditions or after treatment with antibiotics, both types of mice had normal phenotypes.

Not only are food and energy intake and satiety co-regulated by these receptors, but GPR 43 expressed in the intestine also improves glucose tolerance and insulin sensitivity by promoting the secretion of Glucagon-like Peptide-1 (GLP-1) from L cells [34]. Kimura et al. [31] furthermore demonstrated that after activation of GPR 43, the insulin-induced Akt phosphorylation in the white adipose tissue was markedly suppressed. The administration of acetate significantly suppressed insulin-induced Akt phosphorylation in the adipose tissue. These effects were not demonstrated in the muscle and the liver.

Summing up, gut bacteria with the potential to digest soluble complex plant polysaccharides into short-chain fatty acids seem to be lacking in T2 DM patients. In healthy subjects, the higher abundance of these bacteria may cause a higher production of short-chain fatty acids, thus activating G-protein coupled receptors. The activation of such receptors has positive effects in terms of reducing food and energy intake, improving insulin sensitivity via activation of hormones such as GLP-1 and PYY and inhibiting fat accumulation in adipose tissue. A lack of such bacteria may lessen the a.m. effects and further promote accumulation of adipose tissue and insulin resistance.

In 2014, De Vadder et al. [35] demonstrated that propionate and butyrate activate intestinal gluconeogenesis (IGN) via complementary mechanisms. Butyrate activates an IGN gene expression through a cAMP-dependent mechanism, while propionate, a substrate of IGN, activates IGN gene expression via a gut-brain neural circuit involving the fatty acid receptor FFAR3. This had metabolic benefits on body weight and glucose control in normal mice, but these effects were reported absent in mice deficient for IGN, despite similar modifications in gut microbiota composition. The authors concluded that the regulation of IGN is necessary for the metabolic benefits associated with short-chain fatty acids (SCFAs) and soluble fibre. Interestingly, since the bacterial fermentation of dietary fibre also produces large amounts of succinate, the authors recently reported that succinate is also a previously unsuspected bacterial metabolite that improves glycaemic control through the activation of IGN [36].

Concerning further effects, it is noteworthy that a regulation of inflammatory responses by GPR 43 has also been reported in some studies [37,38,39].

**Figure 1:**
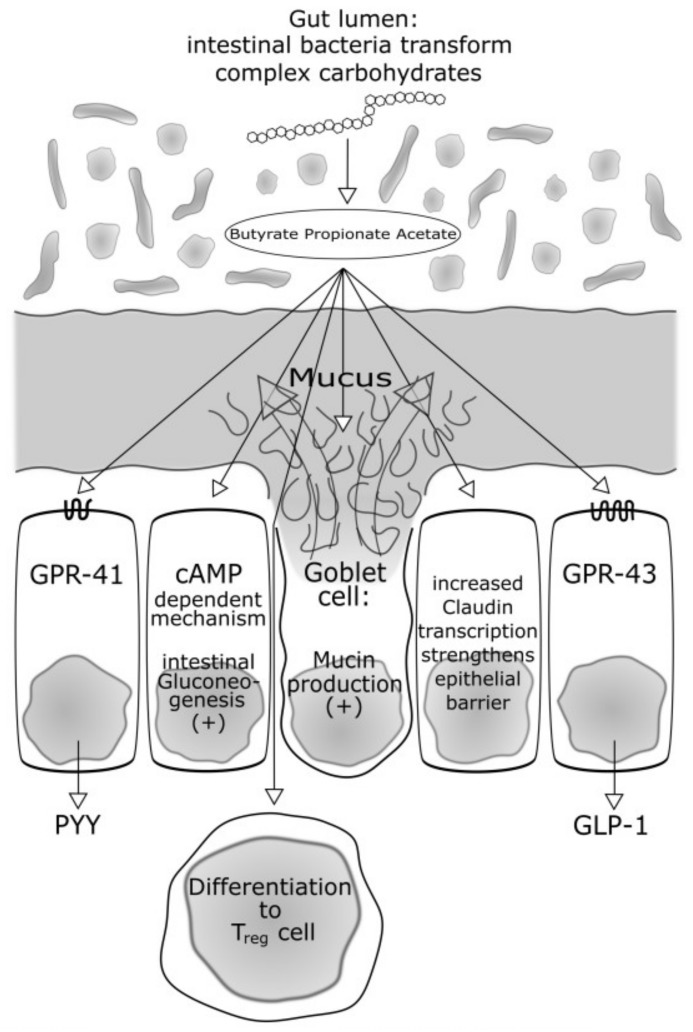
Some of the yet studied effects of short-chain fatty acids (SCFAs): (1) Peptide YY is expressed after SCFA-dependent activation via the G-Protein coupled 41 receptor (GPR-41); (2) Glucagon-like Peptide 1 (GPR-1) is expressed after SCFA-dependent activation via the G-Protein coupled 43 receptor (GPR-43). Glucagon-like Peptide 1 (GLP-1) and PYY inhibit gut motility, reduce appetite and reduce energy harvest; (3) Propionate and Butyrate activate the intestinal gluconeogenesis (IGN) via complementary mechanisms. Butyrate activates an IGN gene expression through a cAMP-dependent mechanism. Propionate, itself a substrate of IGN, activates IGN gene expression via a gut-brain neural circuit involving the fatty acid receptor FFAR3; (4) SCFA promote the formation of peripheral regulatory T cells from naive CD 4+ Cells. It is speculated that SCFAs, especially Butyrate, are inhibitors of some histone deacetylases. An acetylation of Histone 3 promotes the expression of the T regulatory (reg) specific transcription factor Fox P3; (5) Intestinal mucin synthesis. In cultures of human goblet cell-like LS174T cells, Butyrate, as well as propionate, induce an increase in *MUC2* mRNA levels; (6) Protective effects of butyrate for the endothelial barrier. Butyrate decreases the molecular permeability of the intestinal barrier. Butyrate acts through increasing Claudin-1 transcription by facilitating the association between SP1 (transcription factor) and the Claudin-1 promoter.

Figure 1 sums up effects of the SCFAs that are also important in T1 DM which are addressed later.

#### 2.3.2. Microbiome with an Increased Capacity for Energy Harvest in Obesity and in Diabetes

In his “thrifty gene hypothesis”, J.V. Neel [40] proposed a possible genetic predisposition allowing some humans to fatten rapidly and profoundly during times of a good food supply in order to have better chances of surviving during times of famine. This capacity might have outweighed the disadvantage of concurring insulin resistance and the higher risk to develop diabetes. Nowadays, with good food supply in the industrialized countries, the disadvantages of these capacities predominate. Mechanisms to accumulate and store energy may not only lie in the host genome, but also in the actions of the gut microbiota, thus, the metagenome. Turnbaugh's [10] concept of an “obesity-associated gut microbiome with increased capacity for energy harvest” has already been introduced. The concept is supported by the study of Karlsson et al. [24] who demonstrated that in humans, the non-diabetic and the T2 DM bacterial communities had different functional composition. Several pathways were differentially abundant in T2 DM and non-diabetic women. These pathways that showed the highest scores for enrichment in T2 DM metagenomes included starch and glucose metabolism, fructose and mannose metabolism and ABC transporters for amino acids, ions and simple sugars. These findings were later confirmed in studies of Qin and Karlsson [21,24].

#### 2.3.3. Gut Microbiota and Bile Acids

Another interesting piece in the puzzle concerning the effects of the microbiota on energy homeostasis is the observation of Swann et al. [41]: In rats, primary and secondary bile acid profiles in different body compartments (liver, kidney, heart, and blood plasma) are different between conventional and GF and antibiotic (streptomycin/penicillin)-treated rats. Furthermore, the bile acid diversity was lower in GF and antibiotic-treated tissues compared to conventional animals. Bile acids exert important signalling functions and the bile acid profile is obviously influenced by the microbiota. Although the global signalling capacity of bile acids is currently unclear, it is well known that bile acids are natural ligands for a nuclear receptor, the farnesoid X receptor (FXR), and the plasma membrane-bound bile acid receptor TGR5. By activating these receptors, bile acids regulate glucose, lipid and energy homoeostasis [42,43,44,45]. Even though this field warrants further studies, it seems reasonable to assume different effects of different bile acid profiles on energy homeostasis influenced by gut microbiota. There is convincing evidence in animal models. However, human studies are not yet published.

#### 2.3.4. Influence on the Immune System—Low-Grade Metabolic Inflammation (I): Short-Chain Fatty Acids and the Inflammasome

The metabolic syndrome and T2 DM are characterized by low-grade metabolic inflammation [46]. The gut microbiota and its products interact via several mechanisms with the immune system with their interplay yet poorly understood. Such mechanisms are not only investigated in terms of diabetes research, but also for other organ systems. For example, some mechanisms were well-characterized in the airway system of mice [47]: Mice fed a high-fibre diet had increased circulating levels of SCFAs and were protected against allergic inflammation in the lung, whereas a low-fibre diet decreased levels of SCFAs and increased allergic airway disease. Treatment of mice with the SCFA propionate also led to alterations in bone marrow haematopoiesis, which were characterized by enhanced generation of macrophage and dendritic cells.

The aforementioned SCFAs also play a role concerning regulatory T cells which have a key role in limiting inflammatory responses in the intestine. In mice, butyrate—produced by their commensal microorganisms during starch fermentation—facilitated extra thymic generation of T regulatory (T reg) cells. T reg cells are rather anti-inflammatory cells and the authors conclude that the balance between pro- and anti-inflammatory mechanisms is affected by the commensal microbiota and the bacterial spectrum and its capacity to produce SCFA [48].

Inflammasomes are multi-protein complexes that function as sensors of endogenous or exogenous damage-associated molecular patterns. These proteins convert pro-inflammatory cytokines such as interleukin (IL)-1β and IL-18 to their active forms in response to ‘alarm’ signals. Elinav et al. [49] demonstrated that a deficiency of NLRP6 (a gene that encodes proteins of the a.m. multi-protein complexes) in mouse colonic epithelial cells resulted in reduced IL-18 levels and altered faecal microbiota, which was characterized by an expanded representation of the bacterial phyla *Bacteroidetes* (*Prevotellaceae*) and TM7.

In a further study of inflammatory processes in the liver [50], it has been demonstrated that feeding adult mice a methionine-choline deficient diet (MCDD) induces several features of human non-alcoholic steatohepatitis (NASH), including hepatic steatosis and inflammatory cell infiltration. In inflammasome-deficient mice, this feeding caused significantly higher serum alanine transaminase (ALT) and aspartate transaminase (AST) activity by enhanced microvesicular and macrovesicular hepatic steatosis and by the accumulation of multiple immune subsets in the liver from the innate and adaptive arms of the immune system. Interestingly, cohabitation of the mice led to comparable disease features in the wild-type mice. Mice are coprophagic and the results demonstrate that modulation of the intestinal microbiota through multiple inflammasome components is not only a critical determinant of fatty liver disease progression but also other aspects of metabolic syndrome such as weight gain, subclinical inflammation and disturbed glucose homeostasis.

These findings also suggest a modulation of the gut microbiota and its products by the host, and vice versa. However, the findings stem from mice models and its relevance in humans has to be established yet.

#### 2.3.5. Influence on the Immune System—Low-Grade Metabolic Inflammation (II): Endotoxinemia and the Lipopolysaccharides

Apart from the role of SCFAs and the inflammasome on subclinical inflammation characteristic for the disorders of the metabolic syndrome, the lipopolysaccharide (LPS) content of the microbiota may also be a key player. Lipopolysaccharides (“Endotoxin”) are one of the potent virulence factors of Gram-negative bacterial species and have a major role in both acute and chronic infections. This glycolipid is located at the outer membrane of the bacteria, but in the systemic circulation 80–97% of it is bound to the lipoproteins [51]. The “Endotoxinemia” concept was introduced by Cani et al. [52]. In a 4-week study, the authors demonstrated that a high-fat diet in C57bl6/J mice did significantly increase plasma LPS. Because LPS is an important component of lipoprotein and dyslipidaemia is an important feature of metabolic disease, the authors referred to this phenomenon as “metabolic endotoxinemia.” Furthermore, *Bacteroides*-like mouse intestinal bacteria, were significantly reduced in animals fed the high-fat diet. These effects are also achieved by implanting a subcutaneous osmotic minipump and performing a chronic continuous subcutaneous infusion of LPS. Increased fasted plasma glucose and insulin levels as well as insulin resistance were observed. As for metabolic inflammation, the mRNA concentrations of tumor necrosis factor (TNF)-α, IL-1, IL-6, and plasminogen activator inhibitor (PAI)-1 were increased in the liver, as well as in the visceral and subcutaneous depots, and in muscle of high-fat diet-fed and LPS-infused mice.

Antibiotic therapy with ampicillin and neomycin (broad-spectrum antibiotics that are poorly absorbed) was associated with changes in the gut microbiota and endotoxemia during the ingestion of the high-fat diet [53]. Alterations of microbiota and endotoxinemia can also be induced by prebiotics.

Under normal circumstances and in healthy subjects, the translocation of LPS through the gut epithelium is restricted. That said, the studies summed up above suggest that a barrier dysfunction may be inducible by a high-fat diet and expose the host to high levels of translocated LPS. These may induce processes leading to subclinical inflammation and insulin resistance. The study group of Cani could demonstrate [54] that indeed, via a GLP-2 driven mechanism, gut permeability is altered. The gut permeability is controlled by specific tight-junction proteins. ZO (zonula occludens)-1 protein and occludin are proposed to be key markers of tight-junction integrity and a prebiotic treatment increased ZO-1 and occludin mRNA in the jejunum segment in mice under a comparable approach as described above.

As stated in previous chapters, many findings stem from mice models. Its relevance in humans is also yet to be established. Lassenius et al. [55] investigated whether bacterial lipopolysaccharide (LPS) activity in human serum was associated with components of the metabolic syndrome in T1 DM patients. In these patients, a high LPS activity was associated with higher serum triglyceride concentration, earlier onset of diabetes and increased diastolic blood pressure. In a cohort from the FINRISK 97 study, Pussinen et al. [56] reported that both those subjects with prevalent diabetes (*n* = 537) and those with incident diabetes (*n* = 462) had higher endotoxin activity than the nondiabetic individuals and the endotoxin activity was significantly associated with increased risk for incident diabetes. This risk was independent of the other established diabetes risk factors.

Remaining problems, however, are the reliability issue of the endotoxin assays and the yet unsolved issue, whether the LPS detected in humans with the metabolic syndrome are indeed bioactive [57].

#### 2.3.6. Influence on the Immune System—Low-Grade Metabolic Inflammation (III): Metabolic Infection

Metabolic endotoxinemia may not only be a consequence of leakage of lipoproteins and consequently a rise of insulin resistance, proinflammatory cytokines and impaired glucose metabolism, but also of translocation of intestinal bacteria into (adipose) tissue and plasma which maintain low-grade inflammation (“Metabolic Infection”) [58,59]. Amar et al. [60] demonstrated that in mice, after only one week of a high-fat diet (HFD) and before onset of Diabetes, live commensal intestinal bacteria are present in large numbers in the adipose tissue and the blood (the authors quantified bacterial 16S rRNA DNA concentration in different tissues). After 4 weeks of HFD, when the diabetic state was established, the amounts of total bacterial DNA, gram-negative bacterial DNA and *E. coli* DNA were still increasing in the blood. After this 1 week of HFD, the amount of bacterial DNA was increased in mesenteric adipose tissue but not in mesenteric lymph nodes, which occurred after 4 weeks. This bacterial translocation process from the intestine to tissue could be reversed by six weeks of treatment with the probiotic strain *Bifidobacterium animalis* subsp. *lactis* 420. This improved the animals’ overall inflammatory and metabolic status.

The authors also assessed the association between blood microbiota and incident cardiovascular disease in a later publication. In 3936 participants in the D.E.S.I.R. study (longitudinal study with the primary aim of describing the natural history of the metabolic syndrome and its complications), dysbiosis in blood microbiota, defined by a decrease in blood bacterial DNA content and an increase in the proportion of *Proteobacteria* phylum within blood microbiota in the patients with cardiovascular events was observed [61].

## 3. Type 1 Diabetes Mellitus

In contrast to the metabolic syndrome and T2 DM, type 1 diabetes is an autoimmune disease caused by a cellular-mediated autoimmune destruction of the β-cells of the pancreas (T cells). The destruction of the β-cells of the pancreas leads to a consequent insulin deficiency. This form of diabetes accounts for 5–10% of those with diabetes and the patients are typically younger and leaner [62].

However, with the use of insulin therapy and as a result of the global obesity “epidemic”, approximately 50% of patients with T1 DM are currently obese or overweight [63]. That said, some of the pathomechanisms related to overnutrition that have already been described may also account for some T1 DM patients. This part of the review will lay the focus on the specific “autoimmune” aspects of the disorder and the possible impact of the gut microbiota.

The observation of a rapidly rising incidence of T1 DM (and other autoimmune diseases) in the last decades is suggestive of environmental factors contributing to the disorder and cannot be explained by the identified genetic risk variants. The substantial increase of antibiotics usage in medicine and in agriculture over the past 50 years may be such a factor [64]. Other factors may be nutrition, natural birth vs. caesarean section, hygiene status etc. [64]. As stated in the previous chapters about the metabolic syndrome and T2 DM, we will initially focus on a possible altered gut microbiota composition in T1 DM. It needs to be emphasized that these findings demonstrate microbial changes and that further functional studies are needed to prove causality between these kinds of changes and β-cell autoimmunity, which will be described later.

### 3.1. The Role and Composition of Gut Microbiota with Special Regard of Diabetes Mellitus Type 1

In murine models, associations between gut microbiome composition and type 1 diabetes or anti-islet cell autoimmunity have already been reported. The scientist’s favourite pet for the study of T1 DM is the non-obese diabetic (NOD) mouse. Their diabetes has several features comparable to human T1 DM such as immunopathogenesis, genetic susceptibility and responsiveness to environmental factors [65]. In these mouse models, it had already been demonstrated that the probiotic treatment of NOD mice prevented the onset of T1 DM [66] and a low-fat diet together with *lactobacillus* strains also reduced diabetes in NOD mice [67]. Antibiotics are able to prevent the onset of T1 DM in rats that are prone to diabetes (BB-DP rats) [68]. Furthermore, the incidence of diabetes in NOD mice is increasing in a germ-free environment [69]. As for the microbial composition in the gut of BB-DP rats, Roesch et al. [70] reported a higher abundance of *Lactobacillus* and *Bifidobacterium* in a control population of BB-DR (bio-bred diabetes resistant) rats.

One of the first studies in humans with T1 DM was conducted by Giongo et al. [71]. The authors investigated stool samples from four Finnish children who all developed autoimmunity and T1 DM over time and four histocompatibility leukocyte antigen (HLA)-DQ and age-matched children served as controls. The samples were collected at three different points in time, the first about 120 days, the last about 600 days after birth. At the phylum level, the *Bacteroidetes* increased continuingly in between the three sampling points, whereas they decreased in the controls. The *Firmicutes* expressed an inverse pattern. *Clostridiae* increased in the T1 DM children, but decreased in the controls. Although the case number was low, a striking finding was the increase of the *Bacteroidetes* in line with the children developing autoimmune pathology.

De Goffau et al. [72] compared the intestinal microbiota composition in children with at least two diabetes-associated autoantibodies (*n* = 18) with autoantibody-negative children matched for age, sex, early feeding history and HLA-DR risk genotype. The major finding of this analysis was that the *Bacteroidetes* phylum, the *Bacteroidaceae* family and the *Bacteroides* genus were more common in autoantibody-positive children than in autoantibody-negative peers. *Roseburia faecis* was more abundant in autoantibody-negative than autoantibody-positive children whereas *Clostridium perfringens* were more abundant in children with β-cell autoimmunity than in those without. The *Bacteroides* genus was associated with autoantibody positivity. The children with a higher number of autoantibodies had lower numbers of short-chain fatty acid producers than the control children. The authors speculate that the correlation of certain bacterial findings with the number of positive autoantibodies could indicate a role of dysbiosis as a regulator of β-cell autoimmunity in the progression of the autoimmune process towards clinical disease.

Endesfelder et al. [73] sought to determine whether differences are present in the early composition of the gut microbiome in children who developed anti-islet cell autoimmunity. They investigated the microbiome of 298 stool samples prospectively taken up to age 3 from 22 case children who produced anti-islet cell autoantibodies and 22 matched control children who remained islet cell autoantibody-negative in the follow-up. Contrastingly, in this much larger cohort, and after correction for multiple testing, there were no individual bacterial genera that showed significantly different abundances between anti-islet cell autoantibody-positive and anti-islet cell autoantibody-negative children. The microbiome changed markedly during the first year of life, and this was further affected by breast-feeding, food introduction, and birth delivery mode. As possible reasons for the discrepancy with the two previously reported Finnish studies, the authors discuss the differences in sample sizes, the different study design reported by de Goffau et al. [72], and/or possible geographical differences between the German and Finnish children.

In a cohort of 33 infants genetically predisposed to T1 DM, a marked drop in alpha-diversity (the Chao1 alpha-diversity is a measure of the number of distinct microbes in a community) was observed in the T1 DM progressors in the time window between seroconversion and T1 DM diagnosis, accompanied by spikes in inflammation-favouring organisms, gene functions, and serum and stool metabolites. This means that an increased amount of potentially pathogenic bacterial species was detected at that time [74].

However, in the most recent study of de Groot et al. [75], 53 T1 DM patients with a disease duration between 5–16 years (mean 9 years), were compared with 52 healthy controls. In this first observational study in subjects with long-standing T1 DM, the authors reported that the faecal analysis showed decreased butyrate-producing species in T1 DM and fewer butyryl-CoA transferase genes. Furthermore, plasma levels of acetate and propionate were lower in T1 DM, with similar faecal SCFA.

Since the composition of gut microbiota may be different in different geographical regions, different nutritional habits, etc., 15 T1 DM Chinese children (vs. 15 controls) were also examined and the authors reported an imbalance of the faecal microbiota composition, too [76].

A novel approach concerning gut microbiota composition in gut microbiota was reported by Pellegrini et al. [77]. The authors made an attempt to clarify whether changes in the gut microbiota may rather reflect a common “autoimmune milieu” (in other words, a composition of the gut microbiota predisposing for the development and/or the chronification of autoimmune diseases) or if changes are specific between different autoimmune disorders. In another novel approach, the authors did not only evaluate the inflammatory profile, and the microbiome, but also their correlation on the same duodenal biopsies of patients with T1 DM compared with healthy control subjects and patients with celiac disease (CD). The study included 19 individuals with already diagnosed T1 DM, mean age 34 years, mean diabetes duration 20 years, 16 healthy control individuals, mean age 38 years and 19 individuals with CD (untreated) diagnosed at the time of biopsy, mean age 5 years. In measuring the expression of 91 genes related to inflammation (cytokines, chemokine receptors and chemokines) in the gut mucosa, 13 genes were significantly more expressed in patients with CD compared to control subjects. Four genes were more expressed both in patients with CD and in patients with T1 DM compared to control subjects. Ten genes were significantly more expressed in patients with T1 DM but not in patients with CD compared to control subjects. As for the leucocytes infiltrating the duodenal gut mucosa, lymphocytes (CD3-positive cells) in the lamina propria were present in all the groups, their percentage was significantly higher in patients with CD compared with control subjects and patients with T1 DM. The analysis of neutrophil infiltration resulted in a low percentage of positive cells in all biopsies without any significant difference among the three groups.

The composition of bacterial populations was measured using ultra-deep pyrosequencing. The mean bacterial diversity, as estimated by the Chao1 index from the equalized data sets, was not different among the groups, although significant differences in the phyla distribution were observed. The patients with T1 DM showed a reduction in the percentage of *Proteobacteria* and an increase in *Firmicutes.* The phylum of *Bacteroidetes* showed a trend to reduction in patients with T1 DM and patients with CD compared with control subjects. Also, the ratio of *Firmicutes*/*Bacteroidetes* was significantly increased in T1 DM and the CD group. In conclusion, the authors report that the duodenal mucosa in T1 DM shows a peculiar signature of inflammation and a specific microbiome composition. Furthermore, the authors discovered an association between some analysed inflammatory markers and specific taxa. The findings in comparison with the CD group suggest that some changes are specific between different autoimmune disorders.

Summarizing, the results of studies addressing the gut microbiota composition in T1 DM are sometimes conflicting with a trend towards altered composition. The meaning of this and possible autoimmune pathomechanisms are addressed in the following chapter:

### 3.2. Possible Action and Pathophysiology of Gut Microbiota in the Development of Diabetes Mellitus Type 1

#### 3.2.1. Impact of Gut Microbiota on Toll-Like Receptors

Toll-like receptors (TLR) are structures of the so-called innate immune system and belong to a group of Pattern Recognition Receptors. Toll-like receptors are used to recognize structures that occur exclusively on or in pathogens and control a corresponding activation of genes. As a result, the activation of the “antigen-specific acquired immune system” is initiated and modulated. The “toll-like receptors” enable the innate defence system to differentiate between “self” and “not self” [78]. The first attempt to study innate immune sensing in T1 DM in the context of microbial exposure was undertaken in NOD mice genetically deficient of MyDD88, an adaptor protein of multiple TLRs. In a series of experiments, Wen et al. [79] demonstrated that a colonization of these germ-free MyD88-negative NOD mice with a defined microbial consortium (representing bacterial phyla normally present in human gut) attenuates T1 DM. The authors also reported that MyD88 deficiency changed the composition of the distal gut microbiota. An exposure to the microbiota of specific pathogen-free MyD88-negative NOD donors attenuates T1 DM in germ-free NOD recipients, whereas germ-free conditions were not protective. This means that dysbiosis of gut microbiota in MyD88−/−NOD mice resulted in protection from diabetes development. In summarizing these findings, the authors concluded that an interaction of the intestinal microbes with the innate immune system is a critical epigenetic factor modifying T1 DM predisposition and manifestation.

In further studies, the group [80] investigated whether gut bacteria from diabetes-resistant mice could transfer diabetes protection to otherwise highly diabetes-susceptible hosts. They transferred faecal bacteria from diabetes-resistant female MyD88−/−NOD to female NOD/LtJ mice through drinking water for 3 weeks (LtJ means Jackson laboratory and does not refer to a specific feature). The transfer of the gut bacteria of the diabetes-resistant MyD88−/−NOD strain conferred diabetes protection to the NOD mice. In line with the delay in diabetes observed in the mice receiving the MyD88−/−NOD bacteria, there was also a reduction in insulitis in these NOD mice.

That MyD88-negative mice in GF, but not in specific pathogen-free, conditions develop the disease can be understood either by expansion of particular protective bacteria (“specific lineage hypothesis”) or by dominance of negative (tolerizing) signalling over proinflammatory signalling (“balanced signal hypothesis”) in mutant mice. This prompted Burrows et al. [81] to colonize the germ-fee mice with a variety of intestinal bacteria. This proceeding could reduce T1 DM in MyD88-negative (but not wild-type NOD mice), favouring a balanced signal hypothesis. Receptors and signalling pathways involved in prevention or facilitation of the disease remain largely unknown.

#### 3.2.2. Impact of Sex Hormones on the Development of Gut Microbiota Promoting or Preventing Autoimmunity

Maybe less relevant for humans, but interesting from a scientific point of view, is the observation that female NOD mice [65,82] are more prone to develop T1 DM than males. In humans, there is no sex bias in the development of T1 DM; however, in general, other forms of autoimmune disease occur more frequently in women than in men [83]. Transferring the microbiota of adult male NOD mice into weanling-aged female NOD mice is protective against T1 DM [84] and has effects on the microbiota composition in the recipient mice. Transferring the microbiota of adult female NOD mice into the weanling-aged female NOD mice did not yield similar effects. Furthermore, the transfer of the male microbiota also led to a rise in the serum testosterone levels in the recipients.

This phenomenon does also not occur when the testosterone levels in the recipient are neutralized after therapy with flutamide—an androgen-receptor antagonist—as an implant for 60 days. The data suggests that in early life stages there is also a hormone-dependent phase of microbiota development that alters its composition and its ability to promote or prevent mechanisms leading to autoimmunity. The study group supported their hypothesis by further studies [85], and it was confirmed by other groups [86].

#### 3.2.3. Endotoxinemia and the Lipopolysaccharides—Role in Autoimmunity

The biological relevance of LPS has already been described previously in this review. It is well known that T1 DM incidence increases in northern regions, but even in northern regions the incidence varies. e.g., there is a 5–6 times higher incidence in autoimmune disorders and T1 DM in Finland [87] vs. in Karelia (Russia). The DIABIMMUNE study recruited about 1000 infants from Finland, Karelia and Estonia [88] in order to monitor the development of autoimmune disease in these regions. In a subcohort of 74 infants from each of those 3 regions, selected on the basis of similar HLA risk class distribution and matching gender, Vatanen et al. [89] investigated the composition, diversity and stability of gut microbiota in monthly stool samples from an individual from its birth to age 3. The cohort with the highest prevalence of T1 DM autoantibodies was the Finnish. Finnish and Estonian children had higher levels of *Bacteroidetes* throughout the 3-year period, Russian children had higher levels of the phylum *Actinobacteria* during the first year of life. Without going into too much detail, there were strong global differences between the Russian versus Finnish and Estonian microbiota, with the largest differences occurring in the first year and dissipating during the second and third year. Highlighting possible pathomechanisms, the authors demonstrated that the biosynthetic process of LPS, as well as the Lipid A biosynthetic process was different in abundance between the countries. Lipid A is particularly interesting, since Lipid A is the LPS subunit responsible for the immunostimulatory properties of LPS—the Lipid A domain of LPS is responsible for immune signalling through the recognition and activation of the Toll-like receptor [90]. That said, structural changes in Lipid A impact recognition by TLR 4 and influence multiple facets of the immune response. Not only did the authors detect hints that microbial communities in Finnish and Estonian subjects produced more LPS, but they also found out that in all three countries, *E. coli* was a major contributor to Lipid A biosynthesis, but in Finland and Estonia a number of other bacterial species contributed to Lipid A biosynthesis potential, many of which belong to the genus *Bacteroides.* In a further series of experiments the authors detected structural differences between LPS and the Lipid A subunits with different immunogenicity. This led to the conclusion and hypothesis that the more prevalent *Bacteroidetes* species in the microbiota in countries with higher susceptibility to autoimmune disease produce a type of LPS with immunoinhibitory properties. This may somewhat antagonize the higher immunostimulatory properties of LPS and their Lipid A subunit produced by the *E. coli* strains. The phenomenon may preclude an early “immune education” and contribute to the development of autoimmune disease.

#### 3.2.4. Butyrate—Also a Protective Role in Autoimmunity?

Another acquaintance in the tools of the microbiota for the co-regulation of the host’s metabolism and immune processes, is Butyrate.

It is to be expected that dietary factors play an important role in the composition of the microorganisms that colonize the human body in early life [73] and the association of altered gut microbiota with autoimmunity has already been described [71,72]. Mucin synthesis and Butyrate may play a major role. Since there is a lot of literature about possible protective effects of Butyrate against autoimmunity, two shall be addressed in more detail: In an metagenomic study of antibody-positive children already reported in the Giongo paper [71], Brown et al. [91] hypothesized that a consortium of lactate- and butyrate-producing bacteria in a healthy gut may induce sufficient mucin synthesis to maintain gut integrity. In contrast, non-butyrate-producing lactate-utilizing bacteria prevent optimal mucin synthesis, as identified in autoimmune subjects. However, the patient number was low. With the impact of breastfeeding as well as different forms of feeding and the time of their introduction still discussed controversially, Endesfelder [92] investigated samples from the BABYDIET cohort in Germany, presently the largest prospective cohort with detailed dietary protocols [93]. The authors investigated 298 stool samples from 44 children participating in the BABYDIET study for microbiome analysis. 147 samples stemmed from 22 children who developed persistent anti-islet cell autoantibodies at a median age of 1.54 years and 151 samples stemmed from 22 children who remained anti-islet cell autoantibody negative. On average, 6.8 stool samples per child were taken from age 0.24 to 3.2 years and, to analyse microbial communities before the development of the first autoantibody, the authors used samples from children that had at least one probe between the ages of 3 and 9 months. The authors identified 3 main types of microbial communities (C1–3). Community C1 consisted of a significantly larger part of the taxonomic orders *Enterobacteriales* and *Lactobacillales* when compared to C2 and C3 and was the only community that included genera from the order *Bifidobacteriales*. Community C2 constituted mainly of *Clostridiales* and *Erysipelotrichales,* both from the phylum *Firmicutes*. This group also contained several specialists that are characteristic of an adult-like community, such as *Ruminococcus*, *Blautia*, or *Akkermansia*. Community C3 included a large proportion of *Bacteroidetes*. Based on the nutritional protocols of the children, community C1 showed increased abundances in breast-fed individuals and decreased abundances in children who were fed a more complex diet, community C2 revealed the opposite pattern with decreased abundances in breast-fed children and increased abundances in children that were given a more complex diet, whereas no obvious dietary pattern was seen for community C3. Interestingly, there was a subgroup of these children that was dominated by *Bacteroides* abundances compared to two subgroups with low *Bacteroides* and increased *Akkermansia* abundances. The *Bacteroides*-dominated subgroup was characterized by early introduction of non-milk diet, increased risk for early autoantibody development, and by lower abundances of genes for the production of butyrate via co-fermentation of acetate. In contrast to children in the *Bacteroides*-dominated subgroup, children in the *Akkermansia*-dominated subgroups harbour a microbiome that seems to prefer butyrate-production through the co-fermentation of acetate. The beneficial effects of butyrate are that it might improve gut integrity, thus not allowing larger molecules to penetrate the epithelial barrier (“leaky gut”) via mechanisms not completely understood.

The “leaky gut” hypothesis in the context of the development of autoimmunity is supported by several experiments: For example, Bosi et al. [94] investigated 81 subjects with islet autoimmunity (18 preclinical, 28 new-onset and 35 long-term T1 DM) and 40 healthy control subjects with a lactulose-mannitol test consisting of the oral administration of the two sugars and measurement of their urinary excretion. All groups of subjects with islet autoimmunity showed an increase in intestinal permeability to the disaccharide lactulose, indicative of a damaged intestinal barrier. Intestinal mucin production is a key factor in maintaining intestinal integrity. In cell cultures (human goblet cell-like LS174T cells), Burger van-Paassen et al. [95] investigated the mechanisms that regulate butyrate-mediated effects on MUC2 (MUC = Mucin) synthesis. Butyrate, as well as propionate, induced an increase in MUC2 mRNA levels.

Protective effects of butyrate on the endothelial barrier were also reported by Wang et al. [96]. The authors reported that sodium butyrate decreased the molecular permeability of the intestinal barrier in a vivo model. As one mechanism, they identified that butyrate acts through increasing Claudin-1 transcription via facilitating the association between SP1 (a transcription factor) and the Claudin-1 promoter. Another beneficial effect of butyrate may be a direct modulation of immune function [97,98,99]. Several of such effects have been reported. For instance, it has been demonstrated that butyrate inhibits vascular cell adhesion molecule 1 (VCAM-1)-mediated leukocyte adhesion to human endothelial cells [98]. Another mechanism concerning the interaction of butyrate with immune function is modulation of the function of intestinal macrophages: A treatment of macrophages with n-butyrate led to the down-regulation of lipopolysaccharide-induced proinflammatory mediators, including nitric oxide, IL-6, and IL-12. These effects were independent of toll-like receptor signalling and activation of G-protein-coupled receptors, two pathways that could be affected by short-chain fatty acids [99].

In summary, the studies of Endesfelder et al. [92] suggest that an increased availability of butyrate in the intestinal tract (depending on breastfeeding and nutrition) have protective effects against a development of T1 DM related autoimmunity.

Putting together these insights suggests that the gut microbiota may be more involved in the progression from autoimmunity against the beta-cell to T1 DM than in the induction or initiation of the disease process [100].

**Figure 2:**
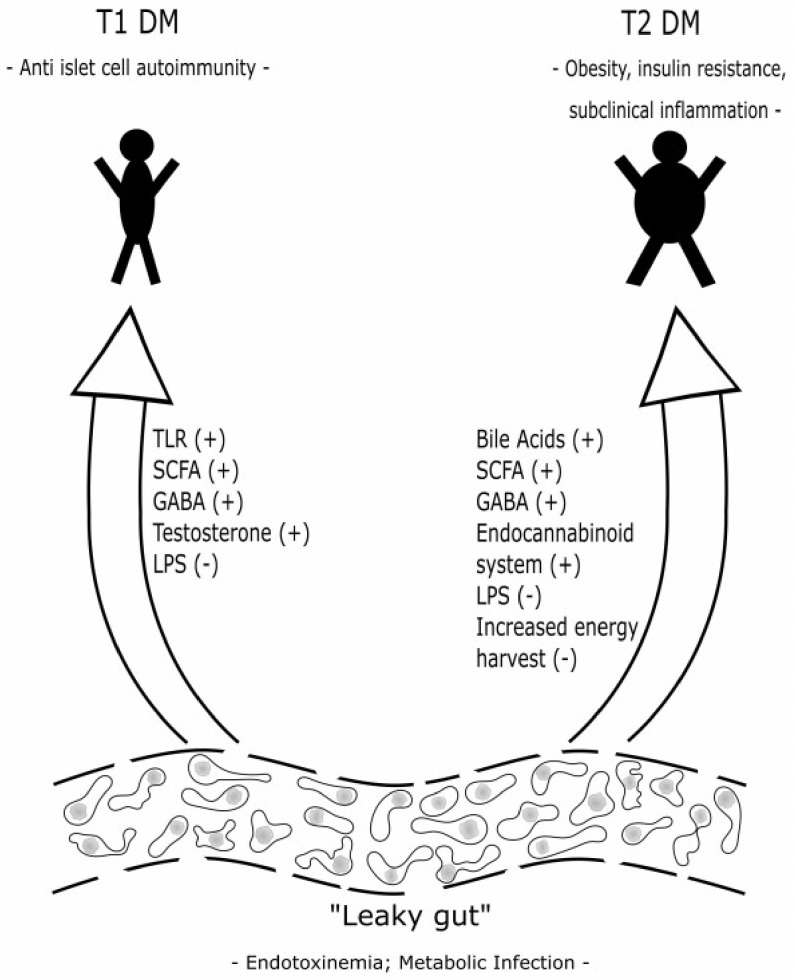
Putative factors in the interaction between gut microbiota and their impact on diabetes mellitus (+: positive; −: negative). Please note that most mechanisms have not been demonstrated in animal models yet. Abbreviations: SCFA: Short-chain fatty acids; GABA: Gamma-aminobutyric acid; TLR: Toll-like receptors; LPS: Lipopolysaccharides.

Figure 2 shows already characterized pathways in the interaction between the human gut microbiome and the host in T1 DM and in T2 DM.

## 4. Therapy

Before the present scientific upsurge in the study of the gut microbiota, therapies were and are still performed that (unintentionally) affect the gut microbiota and may exert some of their beneficial effects via this modulation:

### 4.1. Metformin

Metformin is one of the most widely prescribed oral antidiabetics, since its beneficial effects were reported in the UK Prospective Diabetes Study (UKPDS) [101]. Although this therapy does not intend to modify gut microbiota, and the molecular basis of metformin’s glucose-lowering, weight-reducing and insulin-sensitizing effects is not completely understood, there is a growing body of data suggesting that some effects could be co-mediated by gut microbiota. E.g., in a mouse model, fed with either HFD or normal diet after metformin therapy for 6 weeks, metformin treatment significantly improved the glycaemic profile of HFD-fed mice. HFD-fed mice treated with metformin showed a higher abundance of the mucin-degrading bacterium *Akkermansia* than HFD-fed control mice. In addition, the number of mucin-producing goblet cells was significantly increased by metformin treatment [27]. In humans, the impact of metformin on gut microbiota has recently been reported by Forslund et al. [29]. The authors collected a multi-country T2 DM metagenomic dataset, starting with gut microbial samples from a non-diabetic Danish cohort of 277 individuals within the MetaHIT project and additional novel Danish MetaHIT metagenomes from 75 T2 DM and 31 T1 DM patients, as well as samples from a cohort of 53 female Swedish T2 DM patients along with 92 nondiabetic individuals (43 NGT, 49 IGT) and a subgroup of 71 Chinese T2 DM patients with available information on antidiabetic treatment, as well as 185 non-diabetic Chinese individuals. In all patients, the treatment information was available. For all these 784 gut metagenomes, taxonomic and functional profiles were determined. The fraction of medicated patients was 21% Chinese, 38% Swedish and 77% Danish. As already reported in previous studies, multivariate analysis showed significant differences in gut taxonomic composition between metformin-untreated T2 DM (*n* = 106) patients and non-diabetic controls (*n* = 554), consistent with a broad-range dysbiosis in T2 DM. In the comparison of the T2 DM metformin-treated (*n* = 93) and T2 DM metformin-untreated (*n* = 106) samples, univariate tests of the effects of metformin treatment showed a significant increase of *Escherichia* spp. and a reduced abundance of *Intestinibacter*—the latter fully consistent across the different country datasets—whereas the former is not seen in the Chinese cohort, where diabetics and controls alike are enriched in *Escherichia* spp. relative to Scandinavian controls. Analysis of gut microbial functional potential more generally suggested that indirect metformin treatment effects include reduced intestinal lipid absorption and reduced lipopolysaccharide (LPS)-triggered local inflammation. Taking together the results of the study, it is obvious that concerning further metagenomic studies it is mandatory to report the therapy and this possible impact more cautiously.

### 4.2. Metabolic Surgery

Liou et al. [102] examined whether metabolic surgery alters gut microbiota and whether this alteration itself has metabolical effects: Diet-induced obese (DIO) C57BL/6J mice fed a high-fat diet underwent Roux-en-Y gastric bypass (RYGB) surgery, sham surgery, or sham surgery coupled with caloric restriction. To determine the mode how RYGB affects the distal gut microbiota, the authors performed 16S ribosomal RNA (rRNA) gene sequencing on faecal samples collected before and weekly for 3 months after intervention in the 3 groups. The authors report that RYGB markedly altered the composition of the distal gut microbiota as early as 1 week after surgery, a change that progressed over time and stabilized after 5 weeks. The sham procedure also affected the faecal microbial communities but to a substantially lesser extent than RYGB; and the differences in microbial ecology between the sham group and sham surgery coupled to caloric restriction groups were minimal. These observations suggest that a rearrangement of the gastrointestinal tract by RYGB has a substantially greater effect on the faecal microbiota than either food restriction-mediated weight loss or the limited intestinal disruption caused by the sham procedure. Discriminative features in the RYGB microbiotas were enriched for three distinct taxonomic groups, evident from the phylum level (*Bacteroidetes, Verrucomicrobia*, and *Proteobacteria*) to the genus level (*Alistipes*, *Akkermansia*, and *Escherichia)*, respectively. These changes were similar to those observed in the faecal microbiota of human patients [103,104]. To determine if this composition has anti-obesity properties, the authors inoculated lean, germ-free mice with cecal contents from RYGB donors (and the 2 other groups). The animals exhibited a significant decrease in body weight whereas both SHAM-R and germ-free control animals exhibited no significant weight change. Furthermore, there was a trend toward improved insulin sensitivity, estimated by homeostasis model assessment of insulin resistance (HOMA-IR), and significantly reduced fasting triglyceride levels in the first group. With these observations in mind, the authors conclude that RYGB leads to a specific spectrum of microbiota per se able to improve insulin sensitivity and weight loss, an observation that still needs to be confirmed on human test subjects.

### 4.3. Probiotics

Probiotics and diet are presently relatively safe, non-invasive potential measures in the modulation of the gut microbiota [105]. Earlier studies with the not targeted approach [106] shall not reported here. Since diet is not in the focus of this review (see results of diet reported e.g., in the review of [107]), some of the targeted probiotic approaches against obesity and diabetes are addressed here.

The prevention of metabolic endotoxinemia was a target in numerous studies, frequently probiotic yogurt was applied. Several authors report decreased fasting blood glucose and HbA1c as well as an increased total antioxidant status [108]. As for T2 DM, Akbari and Hendijani recently published a systematic review and metaanalysis [109]. The authors screened 2736 reports and 13 clinical trials met the inclusion criteria. The authors concluded that the administration of probiotics appeared to have a beneficial role in the management of T2 DM, since they significantly decreased fasting blood glucose and HbA1c in diabetic patients. Determinants of the clinical response were participants’ characteristics (e.g., body mass index) and the number and type of probiotic microorganisms used. Another systematic review and meta-analysis published in the same year came to comparable conclusions [110]. The authors additionally state that the findings on HbA1c, anti-inflammatory and anti-oxidative effects of probiotics in the clinical setting remain inconsistent. Further obstacles in the evaluation of probiotic strains also lie in the great number of strains studied, and differences in the detection methodology [111]. All authors agree that the findings in their reviews still imply a need for well-designed clinical studies.

In T1 DM, probiotic approaches may aim more on the modulation of the diabetes risk in stages with HLA-susceptibility or antibody formation than in manifest disease [112].

In animal studies with the BioBreeding rat model of T1 DM Valadares et al. [113] compared the intestinal microbial composition of diabetes-prone and diabetes-resistant animals. The authors reported *Lactobacillus* species negatively correlated with T1 DM development. Two species—*Lactobacillus johnsonii* and *L. reuteri*—could be isolated from diabetes-resistant rats. Diabetes-prone rats administered *L. johnsonii* developed T1 DM at a protracted rate. Interestingly, the analysis of the intestinal ileum showed changes in the native microbiota, host mucosal proteins, and the host oxidative stress response. A decreased oxidative intestinal environment was evidenced by decreased expression of several oxidative response proteins in the intestinal mucosa (Gpx1, GR, Cat). The administration of *L. johnsonii* also resulted in higher levels of the tight junction protein claudin. In a further study on underlying pathology [114], the authors assessed changes in mesenteric lymph node T lymphocyte profiles between Bio-bred Diabetes-prone (BBDP) rats and nondiabetic BBDP rats. However, despite similar levels of T lymphocytes, IL-17A and IL-23R message levels were both 1.5-fold higher in nondiabetic BBDP rats compared with diabetic BBDP rats. The data shows that the gut-associated mesenteric (but not axillary) lymph nodes of nondiabetic BBDP rats have a significant Th17 bias (which means a differentiation towards a more inflammatory cell spectrum) represented by higher levels of IL-17A and IL-23R.

However, the underlying molecular and cellular mechanisms of interaction between immune system and microbiota remain largely unexplored. Although there is evidence that a differentiation towards Th17 cells is important for the development of a healthy immune system, in a K/BxN mouse model of autoimmune arthritis, the introduction of segmented filamentous bacteria (*Clostridiacea* family) into GF animals reinstated the lamina propria Th17 cell compartment, a production of autoantibodies and arthritis rapidly ensued. This data—although in another animal model—more likely suggests an acceleration of autoimmunity [115].

A more familiar mechanism is the action of GLP-1, a gut-derived peptide with systemic action that stimulates postprandial insulin release, suppresses postprandial glucagon, tonically inhibits antroduodenal motility and mediates the postprandial inhibition of antral and stimulation of pyloric motility. Due to these properties, GLP-1 Analogues and drugs inhibiting its degradation (dipeptidyl-dipeptidase-IV (DPP-IV) Inhibitors) are already in clinical use [116]. A β-cell protection of these substances is also under discussion [117]. These different mechanisms and a delivery of GLP-1 via gut microbiota make it an interesting approach in T1 DM, as well as in T2 DM. Consequently, *E. coli* strains were engineered to secrete GLP-1 [118]. The epithelia stimulated by the engineered strains and glucose secreted up to 1 ng/ml of insulin. In the meantime, the concept has evolved to a study [119] in which diabetic rats were fed daily with human lactobacilli engineered to secrete GLP-1(1–37 fragment). The diabetic rats fed GLP-1-secreting bacteria showed significant increases in insulin levels and, additionally, were significantly more glucose tolerant than those fed a parent bacterial strain. These rats developed insulin-producing cells within the upper intestine in numbers sufficient to replace ~25–33% of the insulin capacity of nondiabetic healthy rats. Comparable results were simultaneously reported by other study groups [120]. These approaches sound promising but are presently in the state of animal experiments.

Another, already well-defined target of probiotic therapy is via the short-chain fatty acids acetate, butyrate and propionate. Their beneficial effects, especially of butyrate has already been reported in previous chapters (see also Figure 1). Thus, promising probiotics may contain enteric butyrate-producing bacteria [121]. These are *Roseburia intestinalis, Eubacterium halli* and *Faecalibacterium* spp. A human faecal transplant study [122] that led to an increase of the levels of butyrate-producing microbiota, such as *Roseburia intestinalis* and had beneficial effects on insulin sensitivity is described in detail in the next chapter.

Further proposed targets for a probiotic therapy in obesity and T2 DM include the endocannabinoid system. It is involved in appetite and the energy homeostasis, gut barrier function and has several other properties [123]. The signalling of the system is via the cannabinoid CB 1 and CB 2 receptors. The location of the CB 1 receptor is mainly in the adipose tissue as well as in liver, pancreas and nervous system. An interesting observation was that [124] in mouse models, obese mice treated with a CB 1 receptor antagonist (SR141716A) for 12 days exhibited significantly reduced gut permeability as could be shown by their reduced plasma LPS levels. Changing the gut microbiota using prebiotics decreased the fat mass development in obese mice and the changes in gut microbiota significantly decreased CB 1 mRNA expression in adipose tissues. That said, a modification of the gut microbiota towards lesser CB 1 receptor expression might have beneficial effects concerning the development of obesity and reduce LPS influx into the systemic circulation.

Gamma-aminobutyric acid (GABA) is produced in the metabolic pathways of many *Lactobacillae,* and *Bifidobacteriae* may also be an interesting target in T2 DM and in T1 DM, since in experimental cell-culture studies, it has shown protective effects in β-cells and increases β-cell proliferation and insulin secretion [125,126]. The approaches via GABA and the endocannabinoid system are yet in the stages of cell culture and animal experiments.

### 4.4. Faecal Microbiota Transplantation

In this review, several animal experiments in which gut microbiome was transferred between individuals have already been reported. In humans with Metabolic Syndrome (MS), and with an “intention to treat” approach, the only published study so far is from Vrieze et al. [122]. Vrieze et al. speculated that a rebalancing of the “obesogenic” microbiota in MS patients via infusion from lean donors could have positive effects on the energy metabolism and insulin sensitivity. 18 overweight males (treatment naive) with a BMI about 35 and other features of the MS underwent small intestine biopsies and a bowel lavage through a duodenal tube. One half was randomly assigned to either gut microbiota infusion of lean male donors (BMI < 23) (allogenic group) or reinfusion of own faeces (autologous group) via duodenal tube. The authors reported an improvement of peripheral insulin sensitivity measured using a hyperinsulinemic euglycaemic clamp technique 6 weeks after the allogenic microbiota transfer. As for hepatic insulin sensitivity, they observed a trend toward improvement. As observed in the animal models, too, they also observed an increase of the levels of butyrate-producing microbiota, such as *Roseburia intestinalis*.

These encouraging results prompted the authors [127] to study a larger group of male subjects with MS. Eventually, 12 subjects were included in the group for autologous faecal microbiota transplantation (FMT) and 26 in the allogenic FMT group. 18 weeks after the allogenic FMT, irrespective of single or repeated therapy, no significant effects on hepatic or peripheral insulin sensitivity could be observed any more. The duodenal and the faecal microbiota composition were similar to the baseline. The authors discuss the lack of long-term clinical effects as a development of resilience of the hosts immune system that occurs in combination with the adherence to the previous diet and lifestyle before the experiment.

However, although with a large variation in treatment efficacy, the effects of the pilot study after 6 weeks could be reproduced. In an attempt to predict a responder status, the authors identified the metabolic responders as characterized by a lower initial faecal microbiota diversity. These subjects also had a higher abundance of *Subdoligranulum variabile* and *Dorea longicatena* and a lower abundance of *Eubacterium ventriosum* and *Ruminococcus torques*.

Randomized, placebo-controlled studies on patients with manifest diabetes mellitus are still not published yet.

## 5. Summary and Perspectives

For a medical doctor, the rapidly evolving field of metagenomics and microbiota-host interaction is almost impossible to overlook. New and complex biomedical methods make it even more difficult to understand the issue.

We are just at the tip of the iceberg of understanding the complex interactions between the microbiota and between the microbiota and the host yet. As for obesity and diabetes, several studies addressed the field of a different composition of the gut microbiota in patients with obesity or diabetes versus healthy controls. Although several investigations showed a trend towards a different composition in patients with diabetes, the patient numbers are often low, the results in part contradictory, and the methodology is different. We still lack knowledge in the characterization of a “normal” gut microbiota composition that may vary in different geographical regions, depend on different nutritional habits, sex, age etc.

In an attempt to understand microbiota-induced pathology for the host leading to disease, several putative mechanisms have already been identified. These are an increase in energy harvest, a modulation of free fatty acids, especially butyrate, of bile acids, of lipopolysaccharides, GABA, an impact on toll-like receptors, the endocannabinoid system and “metabolic endotoxinemia” as well as “metabolic infection.”

It is reasonable to assume that further mechanisms will be discovered in the next years. It needs to be kept in mind that the majority of the pathophysiological concepts were established in animal models. Many of the advances in the understanding, and potential development, of microbiome therapeutics have been demonstrated in rodent models and their generalizability to humans, due to the fundamentally different nature of their respective microbiomes, has yet to be tested. Some strains of bacteria have been identified that seem to have predominantly positive effects on the health of the (human) host metabolism. However, several microorganisms and their metabolic features are not known yet. As for the approach with probiotics, these are commonly regarded as safe; however, caution is required—in cases of therapy with *Saccharomyces boulardii/Saccharomyces cerevisiae* (although in these very cases used to prevent antibiotic-associated diarrhoea or to treat recurrent *Clostridium difficile* associated diarrhoea) fungemias with a lethal outcome in severely ill or immunosuppressed patients were reported [128]. It should also be kept in mind that the present scientific upsurge mainly focuses on bacteria and other persistent or occasional inhabitants of the gut microbiome are still neglected. For example, in the case of T1 DM, viruses like the enteroviruses—in particular the coxsackieviruses—have been reported to infect human pancreatic β-cells. The outcome of the infection depends on the strain of the viruses. Other viruses such as the rubella virus or the rotavirus have also been discussed in the aetiology of T1 DM [129].

As for human studies, especially with the concept of FMT in mind, but also transmittable into the probiotic approach, de Groot et al. [130] have recently expressed the unmet needs: For further investigations, we need randomized, placebo-controlled studies designed to unravel mechanisms in the gut-microbiota crosstalk, a thorough recording of the changes in metabolites already identified (e.g., incretins, SFCA, bile acids), the effects on the immune system with a systematic documentation and inclusion of factors such as nutrition, sex, birth mode, geographical factors and medication. This may also help to identify patients who will take profit from the therapy and those that may not. Caution is necessary; Alang [131] recently reported the case of a 32-year-old female with recurrent CDI who underwent FMT from her overweight daughter. The patient presented again 16 months after FMT, and reported an unintentional weight gain of 34 pounds. In an attempt to develop effective and innovative treatments, it has to be kept in mind that yet, we still lack knowledge of the “perfect” donor and recipient profile. Thus far, it is also unknown whether procedures such as FMT from a donor with a desired phenotype may not put the recipient at risk for other diseases. Further problematic issues such as the sustainability of the procedure and infection have to be addressed, too.
